# Association study between drug prescriptions and Alzheimer’s disease claims in a commercial insurance database

**DOI:** 10.1186/s13195-023-01255-0

**Published:** 2023-06-24

**Authors:** Eric Hu, Tong Shu Li, Nathan E. Wineinger, Andrew I. Su

**Affiliations:** 1 Integrative Structural and Computational Biology, Scripps Research Institute, 10550 North Torrey Pines Rd, La Jolla, CA 92037 USA; 2 Scripps Research Translational Institute, La Jolla, CA 92037 USA; 3 Present Address: Scripps Research Translational Institute, La Jolla, CA 92037 USA

## Abstract

In the ongoing effort to discover treatments for Alzheimer’s disease (AD), there has been considerable focus on investigating the use of repurposed drug candidates. Mining of electronic health record data has the potential to identify novel correlated effects between commonly used drugs and AD. In this study, claims from members with commercial health insurance coverage were analyzed to determine the correlation between the use of various drugs on AD incidence and claim frequency. We found that, within the insured population, several medications for psychotic and mental illnesses were associated with higher disease incidence and frequency, while, to a lesser extent, antibiotics and anti-inflammatory drugs were associated with lower AD incidence rates. The observations thus provide a general overview of the prescription and claim relationships between various drug types and Alzheimer’s disease, with insights into which drugs have possible implications on resulting AD diagnosis.

**Supplementary Information:**

The online version contains supplementary material available at 10.1186/s13195-023-01255-0.

## Introduction

Among various forms of dementia, Alzheimer’s disease is considered a particularly debilitating neurodegenerative disease that has had a significant social impact [22]. Development of treatments for AD, drug or otherwise, is coming along but has been slow [12], in large part due to the limited understanding of the pathophysiology of the disease. While there are clear indicators of AD pathology such as amyloid plaque buildup and neurofibrillary tangles, the functional roles of such disease indicators and their contribution to the pathology are still unclear [6]. As an alternative to de novo drug development, there have more recently been considerations to investigate previously approved drug candidates that can potentially be repurposed for the treatment or prevention of AD [5, 9, 13, 17, 25], taking advantage of the reduced time and cost associated with drug repurposing. Over 50 repurposed drugs are already being tested in clinical trials [2], and constantly more potential candidates are emerging from ongoing research.

Medical records, such as insurance and health records, can be likened to a treasure trove of clinical data, with the capability to provide statistical insights on drug use, disease incidence, medical costs, patient demographics, admission rates, and more [14, 26]. The plethora and variety of data can be joined together to provide a correlative analysis which, while perhaps not conclusive, can provide some directions on potential avenues of research. One such application is considering drug-disease relationships within patients for the purpose of identifying or supporting drug-repurposing targets [9, 33]. In this study, we investigate a compilation of insurance records from a US commercial insurance group, with coverage of the group’s members dating back to 2012. The database thus far has been used in analyses such as drug cost comparisons between hospitals and physician offices [24], cost-saving differences between cancer screening methods [23], trends in drug prices [32], opioid prescription trends during the opioid crisis [35], and relationships between COVID and pre-existing conditions [31]. Thus far, the database has yet to be used for observing correlative drug-disease relationships.

In this study, we report the correlations observed between Alzheimer’s disease and drug prescriptions from the health records of the insurance database. Insurance claims data offers the ability to mine for associations in massive databases spanning millions of individuals. Here, we measured drug associations with two disease metrics: (1) AD incidence rate and time to diagnosis and (2) frequency of AD claims as a proxy for disease severity. The analyses conducted here may reveal previously unidentified trends between drug prescriptions and AD diagnosis within a commercially insured population. It may also serve as a reference to those investigating potential repurposing candidates and/or provide additional support for previously identified candidates for the treatment and/or prevention of Alzheimer’s disease.

## Methods and Materials

All member and claim data for the study were accessed through Blue Cross Blue Shield Axis ®, the largest collection of secure commercial claims, medical professional, and cost of care information. The limited dataset of claims data is derived from the independent, locally operated Blue Cross Blue Shield companies across the USA. The database comprises over 400 million claims compiled from over 9 years worth of data (approximately from 2012 to 2021). The derived records are considered primary data sources that include member demographics, claims made during hospital or doctor visits, and pharmaceutical claims for drug prescriptions.

For the purposes of this study, we defined the incidence of a disease based on the ICD-9 code [30] listed as the primary diagnosis within each medical claim. For AD, we used the ICD-9 code 331.0. Prescription drug information within the database was defined according to the National Drug Code (NDC) format. We mapped the NDC IDs to their active ingredients via RxNorm [19]. Thus in this study, a drug was defined as one of the RxNorm (active ingredient) codes mapped in this way. Drug users were defined as members that made at least one drug prescription claim mapped in this way, while non-users were members that never made a claim for the drug in their available coverage history.

**Fig. 1:**
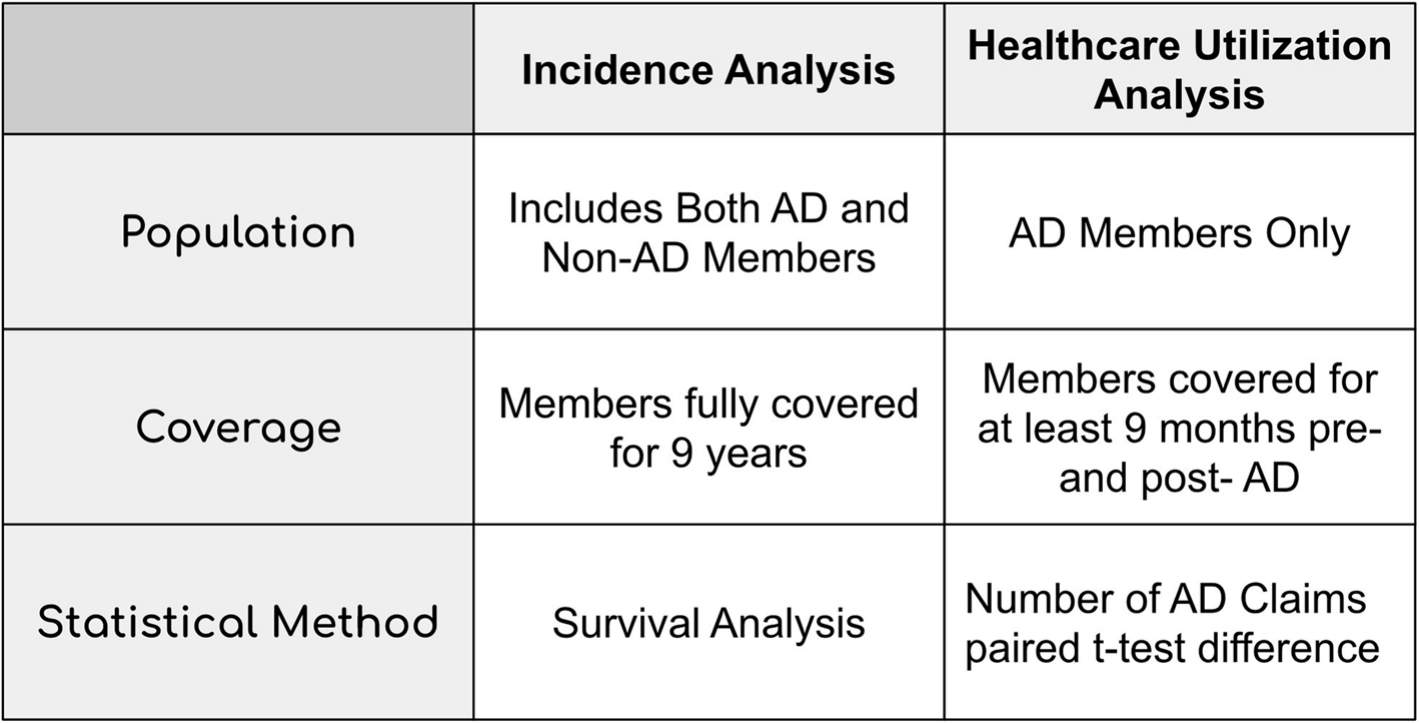
Overview of the statistical analysis methods used in this study

Two primary outcomes were considered: (1) AD incidence rate and time to diagnosis and (2) healthcare utilization related to the disease represented by the number of AD claims. Both analyses were done on a per-drug basis. In summary, for each drug evaluated, members were grouped into users and non-users (Supplementary Methods Figure S1, S3); drug users were propensity matched to those in the non-user group based on age, gender, unique drug usage, and presence of common diseases (via ICD9) (Supplementary Methods Figure S2); and statistical associations between drug use and AD outcomes were conducted. The 200 most common drugs taken by members in the AD group were chosen for the two analyses; additionally, repurposing candidates that were in clinical trials as of 2020 [2] were separately analyzed for AD incidence rate. An overview of the incidence and claims count analyses are summarized in Fig. 1. More detailed explanations for each analysis are provided in the Supplementary Methods.

Inflation of test statistics was controlled using a median quantile adjustment similar to genomic control [7] for the primary analyses. This was achieved by determining the inflation factor (the median observed test statistic relative to theoretical value), scaling test statistics with the inflation factor, and recalculating *p*-values. Afterwards, multiple testing correction was conducted by using the Benjamini/Hochberg method [11].

All analyses were conducted within a virtual, computational environment managed by the Blue Cross Blue Shield Association to ensure proper privacy protection of the sensitive patient data. All modeling and statistical measurements were performed using the statsmodels package within python version 0.10.0 [27]. The IRB protocol number for this project is IRB-19–7372.

## Results

### General statistics and inclusion criteria

The entirety of claims data available spans from 2012 up until the end of 2020 and contains approximately: 113 million distinct members, 751 million inpatient medical claims, 5.2 billion outpatient medical claims, and 3.9 billion pharmaceutical-related claims. There were a total of 143,761 members that had made at least one claim for Alzheimer’s disease (ICD9 code 330.0). Within these members, 92,323 were female and 51,438 were male, and the mean and median age were 86 and 88, respectively. The filters we used in the incidence analysis are described in the following. First is our consideration of age. Alzheimer’s disease disproportionately affects the elderly population that is on average 65 years or older [22]. To improve efficiency in matching and reduce bias from having a large population of younger individuals, an over-encompassing filter was applied where only members that were of age 65 or older at the midpoint of the coverage range (2016) were considered (IE over 60 at 2012, or over 70 at 2021).

Next, we address our decision to use only the individuals with full coverage history. Our concern is the increased matching of pairs with insufficient disease profiles due to a lack of data from a short coverage period. For example, individuals with only a few months of coverage would be matched simply because they made few to no claims during that short time period. It was in our interest to reduce such variability in the matches as much as possible; as a result, we opted to include a filter for the full coverage range of 9 years.

In summary, we only included members that had (1) full coverage history within the database (2012 to 2020), (2) BCBS as their primary provider, and (3) at age 70 + in 2021. These filters limited the number of members with AD to 835. These filters were also applied to members without AD for the analysis and resulted in 101,084 non-AD members.

Within the claim count analysis, the conditions were relaxed since it was already known that all members have made at least one claim for AD. Instead, we opted to find a balance between members having a sufficient amount of coverage information prior to and after AD diagnosis, without reducing the pool of members too much. As a result, we chose members that had at least 9 months of coverage both before and after their AD diagnosis, in which 74,153 members (approximately half of the entire AD cohort) were eligible.

### Alzheimer’s disease incidence rate and survival analysis depending on drug use

**Fig. 2:**
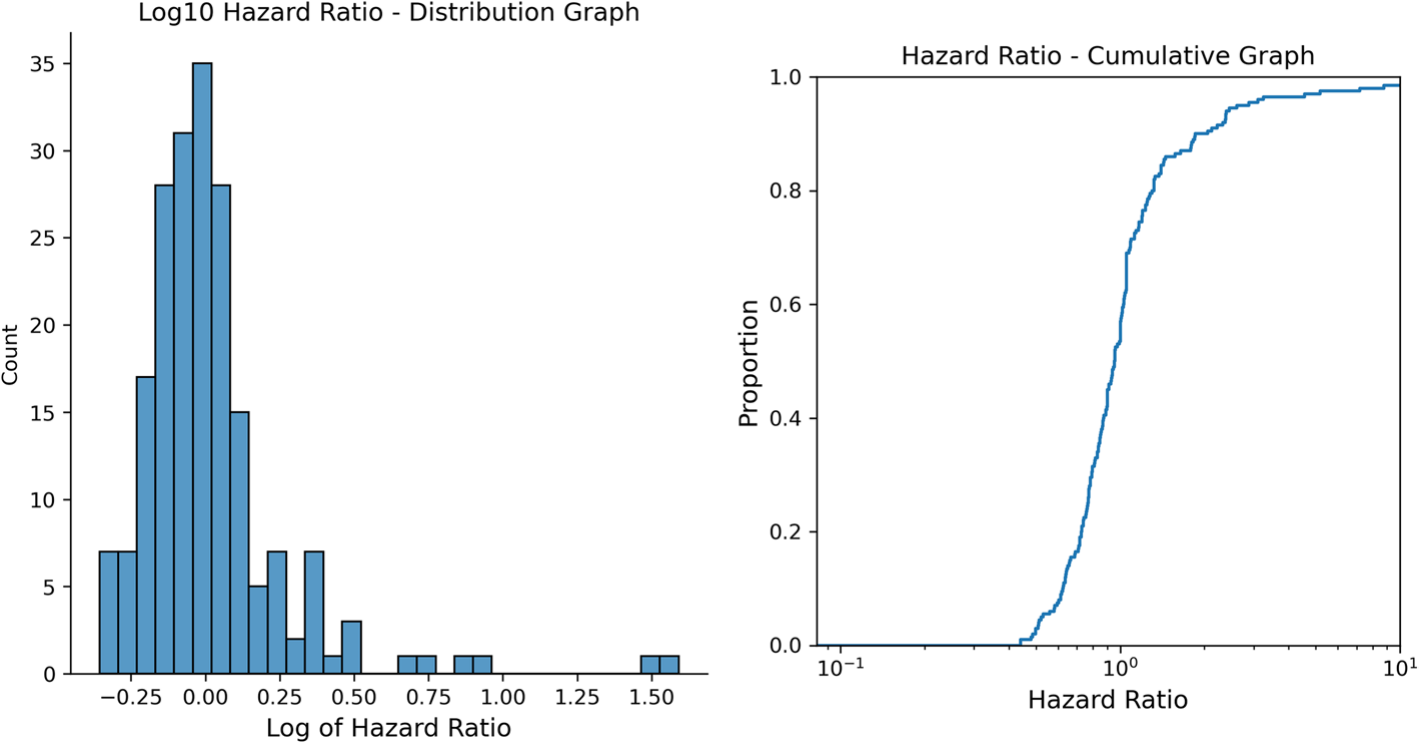
Distribution graph (left) and cumulative graph (right) of the log hazard ratio. Median hazard ratio is 0.95

**Fig. 3:**
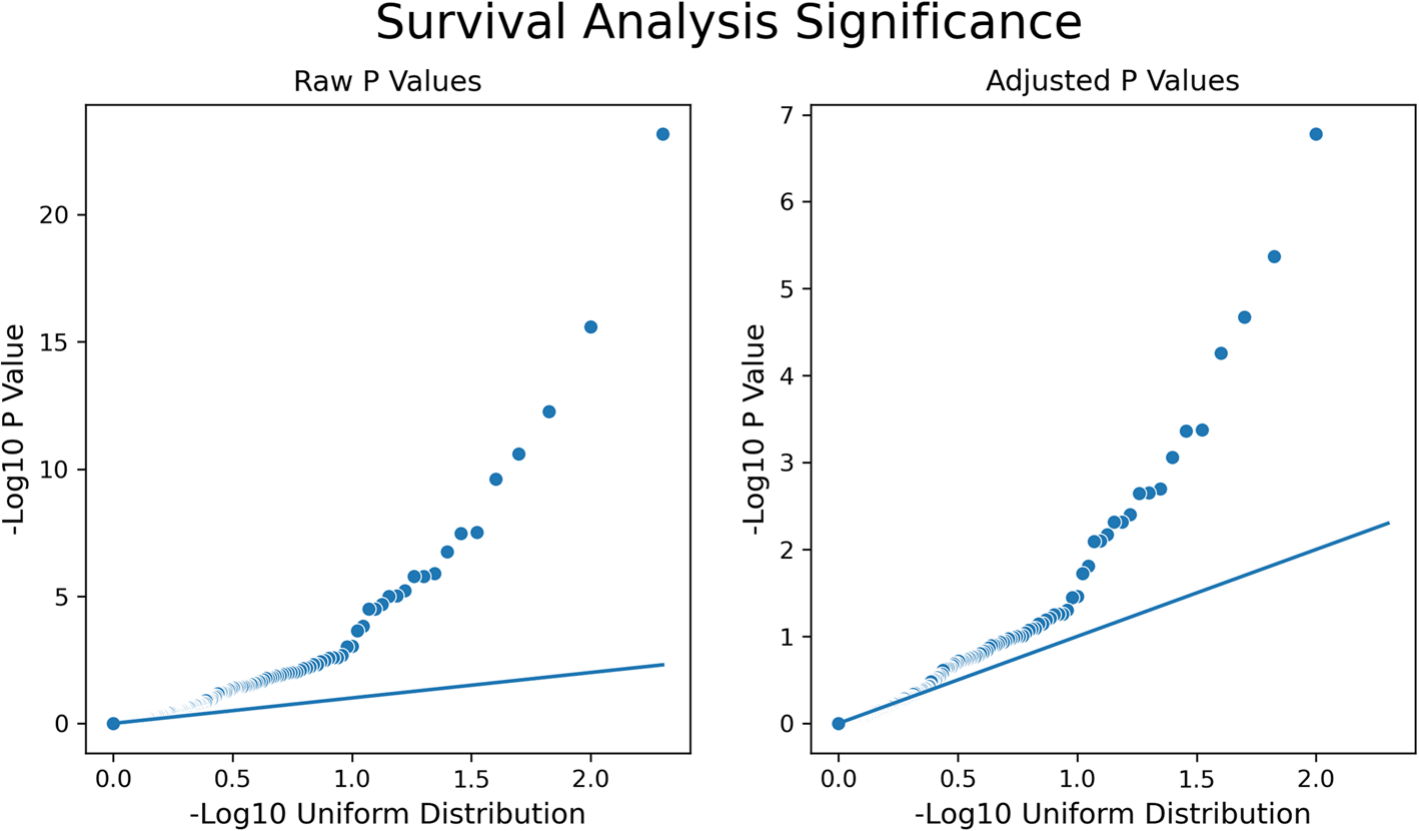
QQ Plots for raw and adjusted *P* values

Survival analysis was conducted on propensity-matched pairs for the top 200 drugs taken by AD patients based on the BCBS database records. The log hazard ratio distribution and the hazard ratio log cumulative graph are shown in Fig. 2. Figure 3 displays the QQ plots for raw and adjusted *p*-values from the survival analysis on the negative log 10 scale. The median hazard ratio for all drugs was 0.95. The inflation factor for the adjusted *p* value was approximately 2.46. We identified 22 drugs having an association with AD incidence with adjusted *p* < 0.05 in our survival analysis, 15 with a decreased risk, and 7 with an increased risk (Table 1).

**Table 1:** Drugs where the inflation-adjusted *P* value was less than 0.05

Drug name	Hazard ratios	Raw *P* value		Lower CI		Upper CI		Total matched pairs			Drug users with AD	Non-users with AD		Inflation-adjusted *P* value	Corrected *P* value		Drug description
**Drugs with low hazard ratios**																	
*Amoxicillin*	0.44	2.55E − 16		0.36			0.54		27,570	144		325	1.67E − 07		1.67E − 05		*Antibiotic*
*Azithromycin*	0.51	2.51E − 11		0.42			0.62		27,276	152		295	2.11E − 05		1.05E − 03		*Antibiotic*
*Methylprednisolone*	0.51	2.48E − 10		0.41			0.63		24,723	132		259	5.50E − 05		2.20E − 03		*Inflammation*
*Doxycycline*	0.52	3.41E − 08		0.41			0.66		21,268	110		210	4.36E − 04		1.25E − 02		*Antibiotic*
*Prednisone*	0.58	1.73E − 07		0.48			0.71		26,539	150		256	8.66E − 04		2.17E − 02		*Inflammation*
*Sodium sulfate*	0.53	1.25E − 06		0.41			0.69		22,539	90		169	2.01E − 03		4.11E − 02		*Laxative*
*Clavulanate*	0.60	1.66E − 06		0.49			0.74		26,156	143		237	2.26E − 03		4.11E − 02		*Antibiotic supp*
*Cephalexin*	0.63	9.70E − 06		0.51			0.77		23,061	149		236	4.81E − 03		6.93E − 02		*Antibiotic*
*Magnesium sulfate*	0.44	9.90E − 06		0.31			0.63		12,507	42		95	4.85E − 03		6.93E − 02		*Electrolyte*
*Acetaminophen*	0.64	3.15E − 05		0.52			0.79		23,245	140		219	7.98E − 03		9.42E − 02		*Analgesic*
*Hydrocodone*	0.66	3.18E − 05		0.54			0.80		27,112	165		250	8.01E − 03		9.42E − 02		*Opioid*
*Ibuprofen*	0.60	1.48E − 04		0.46			0.78		17,714	86		144	1.56E − 02		1.73E − 01		*Inflammation*
*Promethazine*	0.62	9.24E − 04		0.47			0.82		13,467	80		128	3.47E − 02		3.41E − 01		*Antihistamine*
*Benzonatate*	0.65	9.89E − 04		0.50			0.84		17,534	97		149	3.58E − 02		3.41E − 01		*Cough*
*Fluticasone*	0.72	2.10E − 03		0.58			0.89		22,056	148		206	4.99E − 02		4.47E − 01		*Steroid*
**Drugs with high hazard ratios**																	
*Donepezil*	30.70		6.85E − 24		15.78		59.76		1067	235		9	0.00E + 00			0.00E + 00	*Treats AD*
*Memantine*	38.97		5.39E − 13		14.41		105.39		544	132		4	4.25E − 06			2.84E − 04	*Treats AD*
*Quetiapine*	7.19		3.09E − 08		3.58		14.46		1004	63		9	4.18E − 04			1.25E − 02	*Antipsychotic*
*Sertraline*	2.39		1.62E − 06		1.67		3.42		4710	102		43	2.24E − 03			4.11E − 02	*SSRI/anxiety*
*Trazodone*	2.37		6.08E − 06		1.63		3.45		5524	92		39	3.94E − 03			6.56E − 02	*Depression*
*Escitalopram*	3.26		2.08E − 05		1.89		5.61		3830	55		17	6.68E − 03			8.91E − 02	*SSRI/anxiety*
*Mirtazapine*	3.11		2.31E − 04		1.70		5.68		1544	43		14	1.89E − 02			1.99E − 01	*Depression*

Top table displays the drugs with low hazard ratios, while the bottom table presents drugs with high hazard ratios. Each row displays the drug name, hazard ratios and the 95% confidence intervals, calculated *p*-values (raw, inflation-adjusted, and corrected), total number of pairs and the number of members with AD in each group, and a brief description of the drug

Out of 58 repurposed drug candidates that are in clinical trials for the treatment of AD as of 2020 [2], we found 25 drugs where (1) prescriptions for the drug were present with members that had made claims for AD within the BCBS database, providing evidence of use within the member cohort, and (2) drug users were able to be matched to nonusers during propensity matching. The specific candidates were separately compared from the general analyses of the 200 drugs. *P*-value adjustment and correction were not performed due to the lower number of candidates. Table 2 shows the most significant candidates (*p* < 0.05) that were present in the drug list, as well as their survival analysis statistics. A list of all the drugs that were tested can be found in the Supplementary tables. Overall, only 8 of the repurposed drug candidates were shown to have any significant association with AD incidence within the BCBS database.

**Table 2:** Drugs that were found to be significant (raw *p* < 0.05) out of clinical trial candidates available for survival analysis

Drug name	Hazard ratios	Raw *P* value	Lower CI	Upper CI	Total matched pairs	Drug users with AD	Non-users with AD	Trial	Drug description
**Clinical trial candidates with low hazard ratios**									
*Valacyclovir*	0.560	4.72E − 03	0.37	0.84	8519	37	66	Phase II	*Antiviral*
*Montelukast*	0.617	1.39E − 02	0.42	0.91	7254	42	68	Phase II	*Inflammation*
*Losartan*	0.727	3.24E − 02	0.54	0.97	13,399	78	107	Phase III	*Diabetes*
**Clinical trial candidates with high hazard ratios**									
*Escitalopram*	3.258	2.08E − 05	1.89	5.61	3830	55	17	Phase III, phase I	*Anxiety*
*Mirtazapine*	3.105	2.31E − 04	1.70	5.68	1544	43	14	Phase III	*Depression*
*Levetiracetam*	4.034	5.28E − 03	1.51	10.75	877	20	5	Phase III, phase II, European	*Seizures*
*Levodopa*	3.435	1.53E − 02	1.27	9.31	727	17	5	European	*Parkinson’s*
*Eszopiclone*	3.009	3.29E − 02	1.09	8.28	1033	15	5	Phase III	*Insomnia*

Table is split between candidates with a hazard ratios less than 1 and those with a ratios greater than 1

### Healthcare utilization related to AD as represented through claim count analysis within the AD-specific population

We next examined the association between drug prescription claims and the number of AD claims. According to a number of studies, insurance claim count data can be considered a proxy for disease severity [16, 20, 28]. Although differences in AD-related claim counts between drug users and non-users may simply reflect differences in health care utilization, we also believe that strong associations may indicate a drug’s potential influence on the progression of AD.

**Fig. 4:**
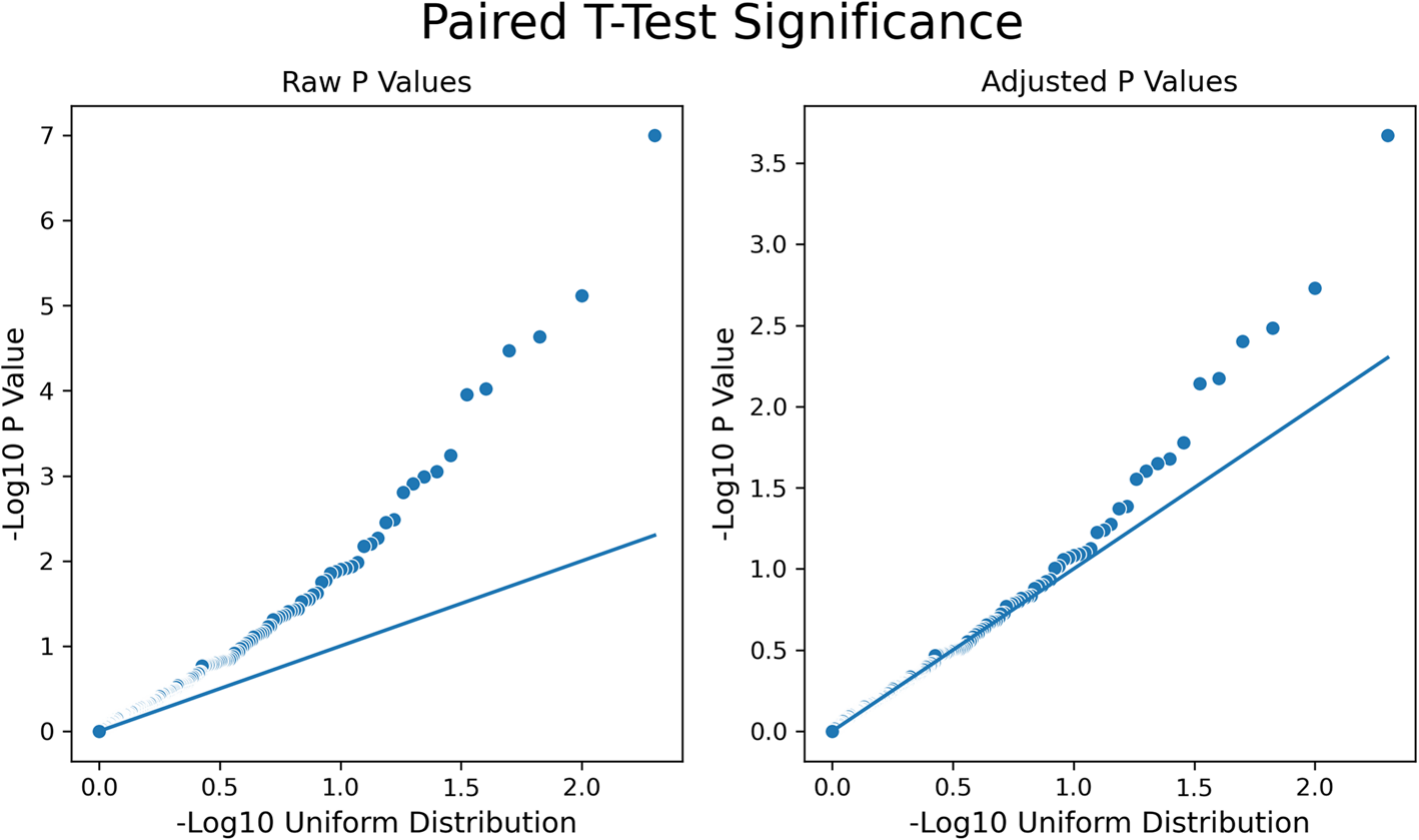
QQ plots for raw and adjusted p-values from the claim count analysis

Propensity-matched drug users and non-users were compared for the top 200 drugs taken within members that had made at least one claim for Alzheimer’s disease. The total number of Alzheimer’s disease-related claims made within the 9 months after the first claim was tallied for the member pairs, and the paired* t*-test statistic and *p* value significance were calculated. Figure 4 shows the QQ plots of *p* value significance (raw, adjusted, and corrected) for the 200 drugs in the analysis. Table 3 portrays the drugs considered significant based on adjusted *p*-value (adjusted *p* < 0.05), indicating that drug users and non-drug users have a non-trivial difference in the number of AD claims made. Overall, this consists of 9 negative and 4 positive paired *t*-test statistic results that were deemed significant. All *p*-values calculated are provided in the table for reference; only one drug (quetiapine) was considered significant when observing the corrected *p*-value.

**Table 3:** Drugs with claim count differences where adjusted *P* value is lower than 0.05

Drug name	Total matched pairs	Drug users—mean AD claim count	Non-users—mean AD claim count	Paired *T*-test statistic	Raw *P* value	Inflation-adjusted *P* value	Corrected *P* value	Drug description
**Decreased healthcare utilization**								
*Penicillin V*	2635	3.93	4.63	− 4.23	2.34E − 05	3.28E − 03	1.98E − 01	*Antibiotic*
*Donepezil*	22,005	4.16	4.44	− 4.15	3.40E − 05	3.96E − 03	1.98E − 01	*Treats AD*
*Sodium sulfate*	6218	4.03	4.51	− 3.90	9.55E − 05	6.68E − 03	2.40E − 01	*Laxative*
*Clavulanate*	10,721	4.16	4.52	− 3.45	5.72E − 04	1.66E − 02	4.75E − 01	*Antibiotic supp*
*Albuterol*	9806	4.18	4.53	− 3.32	8.95E − 04	2.09E − 02	4.96E − 01	*Asthma*
*Sodium chloride*	5789	4.03	4.43	− 3.23	1.24E − 03	2.48E − 02	4.96E − 01	*Electrolyte*
*Celecoxib*	2629	4.01	4.60	− 3.17	1.56E − 03	2.79E − 02	5.06E − 01	*Inflammation*
*Amoxicillin*	18,298	4.22	4.45	− 2.94	3.28E − 03	4.10E − 02	6.52E − 01	*Antibiotic*
*Potassium chloride*	11,608	4.20	4.45	− 2.92	3.50E − 03	4.24E − 02	6.52E − 01	*Electrolyte*
**Increased healthcare utilization**								
*Quetiapine*	5379	5.12	4.36	5.33	1.01E − 07	2.14E − 04	4.28E − 02	*Schizophrenia*
*Valproate*	1643	5.30	4.19	4.48	7.63E − 06	1.86E − 03	1.86E − 01	*Seizures*
*Lorazepam*	7199	4.82	4.36	3.87	1.11E − 04	7.21E − 03	2.40E − 01	*Seizures*
*Trazodone*	5538	4.73	4.29	3.29	1.02E − 03	2.24E − 02	4.96E − 01	*Anti-depressant*

Decreased healthcare utilization, where drug users have fewer claims than non-users, is on the top table; increased healthcare utilization, where drug users had more claims than non-users, is displayed in the lower table

## Discussion

The current study is an exploratory analysis that identifies associations between drug treatment and Alzheimer’s disease in a large insurance claims database. We found that antibiotics, antiviral, and anti-inflammatory drugs had low hazard ratios in our study. Notably, very significant drugs with lower hazard ratios consist of common antibiotics and anti-inflammatory drugs, particularly for those where the corrected P is less than 0.05. A number of ongoing studies have suggested that inflammatory response in the brain is one of the key factors that lead to AD [1, 21]. The observation that taking anti-inflammatory medication results in a lower incidence of AD can be supportive of this association. Likewise, microbial and viral infections have also been associated with a higher risk of AD [4, 13]. The lower hazard ratio from the use of antibiotics and antiviral medication, therefore, could be indicative of a possible protective effect leading to reduced incidence of AD overall. Overall, the results may warrant further investigation into these drug categories to more clearly elucidate if they can have a preventive influence for Alzheimer’s disease.

Our analysis also shows drugs that treat mental illness have high hazard ratios and more claims for drug users. The significant drugs seen with high hazard ratios are those used to treat AD itself or other mental illnesses. This is also seen with the repurposed candidates in clinical trials, where the higher HRs trend towards drugs that are typically used to treat neurological illnesses, as well as the claim counts, in which the significant drugs with a positive paired *t*-test statistic are also related to the treatment of mental illnesses. It is key to note that these trends are unlikely due to the effect of the drug use, but rather that the diseases themselves were not considered in the feature selection during propensity matching. It is particularly notable that the drugs donepezil and memantine, which are primarily used in the treatment for AD, are prescribed prior to the diagnosis of AD within the drug user group. One likely reason for this case is that these drugs are being prescribed for MCI and other forms of cognitive decline prior to the onset and eventual diagnosis of Alzheimer’s disease itself, although studies have shown that the efficacy of such drugs is modest at best [29, 34]. Other medications provide more intriguing insights into the connections between AD and a variety of other mental illnesses such as dementia, schizophrenia, anxiety, and depression. Relationships between these conditions and Alzheimer’s have been seen before, where a number of these conditions result in a higher risk of AD [8, 15, 18]. Furthermore, misdiagnosis for the early stages of AD as a different disorder may be a cause of the associations seen here,misdiagnosis is prevalent for Alzheimer’s disease [10], which could be reflected in the candidates with higher hazard ratios seen in the study.

There are a number of important limitations to this study. First, the misdiagnosis and underdiagnosis of AD could affect the analysis results. Our analysis relied on a single ICD-9 code (331.0) to define AD-positive individuals. While including ICD-9 codes for other dementias could reduce the effect of underdiagnosis, our analysis conservatively defined AD positivity to avoid reporting biased results.

Second, our propensity score model had a finite limit on the number of patient features that could be used in our matching procedure. The model was primarily based on demographic data and individual disease status (also derived from the insurance claims data), which captured a broad cross-section of features for propensity matching. While the addition of more features could undoubtedly improve matching, our analysis was limited by practical considerations of compute requirements and generalizability.

Next, the filters that were applied for the incidence analysis merit discussion. Most notably, full coverage filtering introduces the possibility of survival bias, where only individuals that have “survived” (in this case, still insured by the current group). However, using partial instead of full coverage filters resulted in high inflation of the test statistic and overall predisposition of drugs towards lower hazard ratios (see all Supplementary Results figures). Hence, for both consistency and reliability, our analysis considers individuals with the full coverage range, which reduces the number of poorly matched pairs with incomplete coverage data. Consequently, the average life expectancy after AD diagnosis is approximately 8.3 years [3]; therefore, it is reassuring that the range observed would expect to cover many of the patients that end up being diagnosed within the specified time frame and likely reduce the influence of survival bias.

Finally, there are limitations originating directly from the dataset itself. These include, but are not limited to, (1) prevalence of younger and working demographic population for commercially insured members may bias the cohort to a more healthy deposition; (2) healthcare utilization could simply be a result in better use of the healthcare system rather than severity; and (3) drug prescriptions do not guarantee the actual use of the drug by the patient, or vice versa where the patient may use a particular drug without making a prescription claim.

In conclusion, the observational study described by this manuscript can be considered as a compilation of the associations between the use of commonly prescribed drugs and Alzheimer’s disease within an insured population. Most notable is how observations made in this study can be confirmed with other experimental studies. While the mechanisms behind neuroinflammation leading to AD had been established in experimental settings and are still an active field of study, and the comorbidities of AD with other mental illnesses had been observed before in clinical settings, it is interesting to observe that the trends carry over in an observational study of a commercially insured population. In both cases, where antibiotics were associated with a lower incidence of AD and mental illness drugs associated with a higher incidence of AD, it was both unexpected (assuming the null hypothesis) and reassuring that there is mechanistic reasoning behind the results. In future studies, we hope to further explore these drug-disease associations through secondary datasets and mechanistic validation. Overall, the results from the analysis are provided with the hopes of providing direction and furthering progress on the complex task of understanding, treating, and preventing Alzheimer’s disease.

## Supplementary Information

**Additional file 1: Supplementary Methods. Figure S1.** Diagram representation of the sorting process based on coverage history. **Figure S2.** A pictorial representation of the propensity matching process. **Figure S3.** Selection of segmented portions from non-users based on coverage during the claim count analysis.

**Additional file 2: Supplementary Results. Figure 1.** Log 10 Hazard Ratio Distribution Graph from the survival analysis of partial coverage individuals. **Supplementary Results Figure 2.** Log 10 Hazard Ratio Cumulative Graph from the survival analysis of partial coverage individuals. **Supplementary Results Figure 3.** QQ plot for raw *P* values from the survival analysis of partial coverage individuals.

**Additional file 3.** Raw Data.

## Data Availability

Supporting Data (Results Tables) will be provided as Supplementary Material.
