# Supplementary material for: Association study between drug prescriptions and Alzheimer’s disease claims in a commercial insurance database
Source: Alzheimers Res Ther. 2023 Jun 24;15:118. doi: 10.1186/s13195-023-01255-0 (PMC10290352; doi:10.1186/s13195-023-01255-0)
Supplement: Supplementary file 1 — Additional file 1: Supplementary Methods. Figure S1. Diagram representation of the sorting process based on coverage history. Figure S2. A pictorial representation of the propensity matching process. Figure S3. Selection of segmented portions from non-users based on coverage during the claim count analysis. [file 13195_2023_1255_MOESM1_ESM.docx]

**Supplementary Methods**

*Alzheimer’s Disease Incidence Analysis*

In our analysis of AD incidence, only members that were fully covered by BCBS from 2012 to 2021, the total available coverage history in the database, were considered. Furthermore, since AD is a disease that affects the older population, we only included individuals at least 70 years of age in 2021. Members were also filtered based on whether BCBS was their primary provider since members may have other insurance providers (such as Medicare); primary provider status is important since AD claims made to other providers may not be recorded in the BCBS database, resulting in uncertainty in disease status.

Members were considered part of the AD group if they had made two or more claims with Alzheimer’s Disease as the primary diagnosis. Members without any AD claims were considered non-AD members. The incidence analysis is done on a per-drug basis, and our interest is the possible influence of drugs and diseases on AD incidence. For each drug, we created drug user / non-user groups depending on the presence of drug prescription claims. Within the AD group members, only drug and non-AD disease claims up until the first AD claim were used for this analysis, since post-AD claims would not have an influence on the initial AD diagnosis. Meanwhile, all drug and disease claims were used for the non-AD group.

.


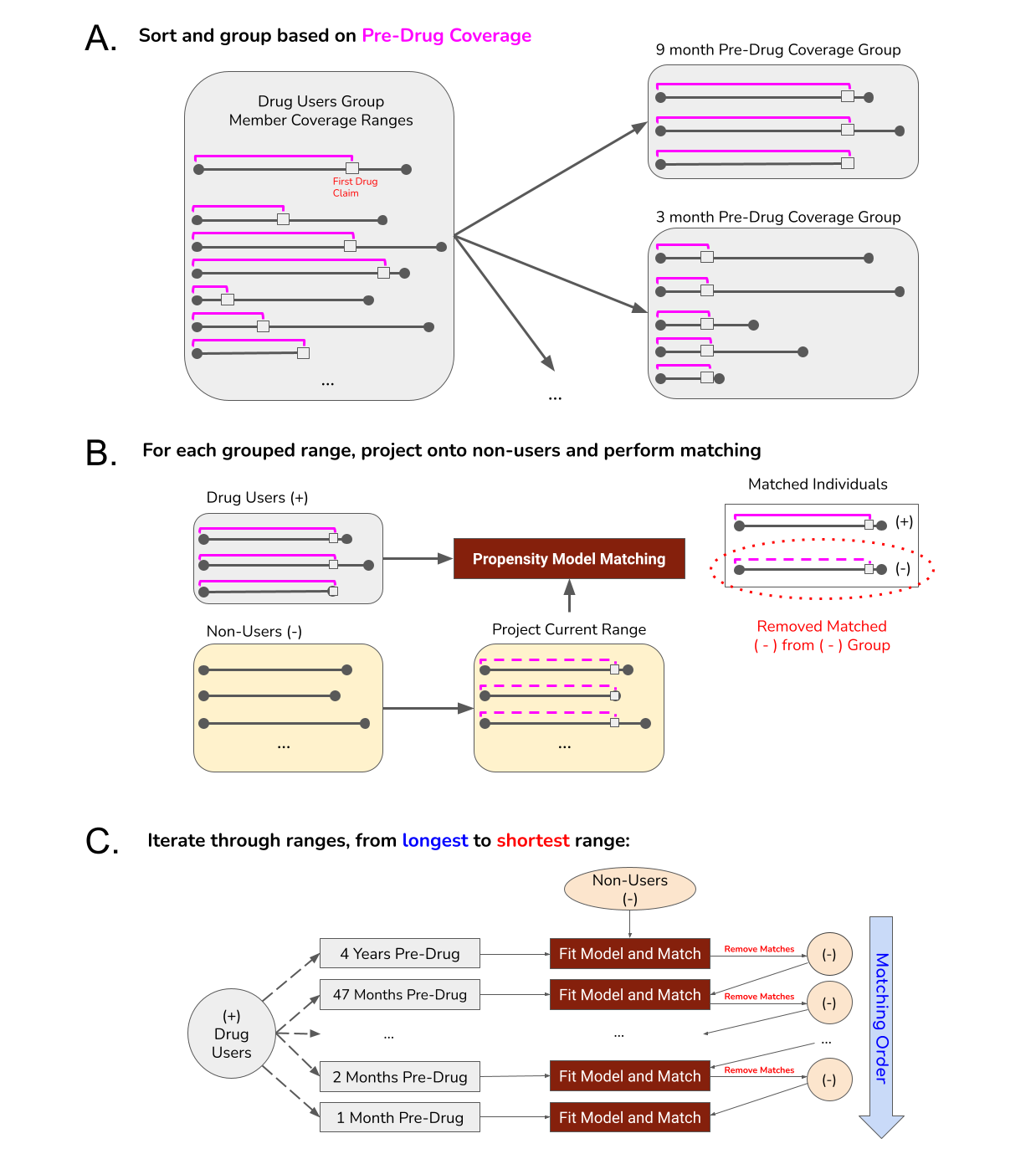


Figure S1. Diagram representation of the sorting process based on coverage history A. The drug user group is binned into coverage ranges based on the length of coverage history available prior to the first instance of the drug prescription claim. B. The same range is projected onto all non-users, which will then be used during propensity matching. C. The process is repeated for every coverage range, starting with the individuals with the longest coverage history.

The data on disease history is variable between drug users depending on the amount of coverage each user has prior to drug initiation date, designated by the users’ first instance of a drug claim. To address this issue, drug users were binned based on the amount of coverage (in months) prior to drug initiation date, and a separate propensity model was developed for each pre-drug coverage history bin (Figure S1). Through this, both the drug and non-drug users were matched on a disease history for the same number of months. Individuals with the longest history are matched first, since more data is provided within these individuals. For each coverage range, a propensity scoring model was fitted using logistic regression based on all available positive and negative drug cohorts.

For every coverage range bin, a logistic regression model was trained and 1 to 1 propensity matching based on the model was conducted (Figure S2). The regression model between users and non-users was trained based on the following features: (1) sex, (2) age at the date of the drug user’s drug initiation date, (3) disease history up until the drug initiation date, based on presence of claims for the top 50 common disease codes between all members in the study, (4) total unique drugs taken by the individual, not counting the current drug. Training data included all drug users that had the coverage history for that available range, and all non-drug users using the projected target coverage range.


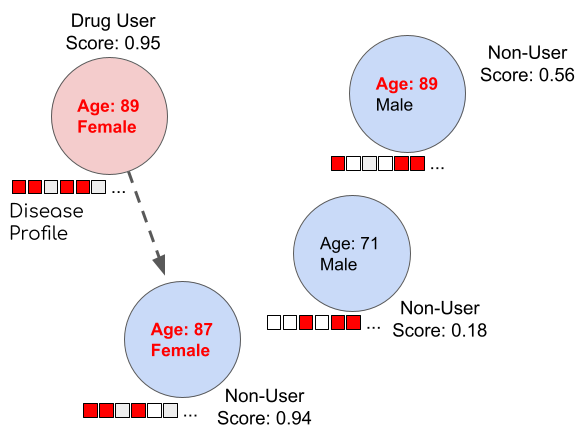


Figure S2. A pictorial representation of the propensity matching process. For every drug user, a non-user with a similar score is chosen for a 1:1 matching. The features that the score is dependent on includes age, gender, common disease profile, and overall unique drug usage.

Following training, individuals were scored based on the trained model, and the non-drug member with the closest score was paired to each drug member through the use of a greedy matching algorithm. Every drug user is matched to a non-user if found within score matching threshold distance of 1% probability, or is omitted if there is no suitable match. After matching, the matched non-users for the current coverage range were removed from the overall pool of non-users, and the matching for the next range was performed with the remaining non-users (Figure S1, part C). Thus, for every drug and each available coverage range, we obtained the members from the drug positive cohort paired with individuals from the drug negative cohort, with the negative members’ coverage projected on the same range as their matched pair.

In our survival analysis, an event is defined as the first incidence of Alzheimer’s Disease. The survival analysis was performed on the propensity matched drug user/non-user groups in which the designated event is the first AD claim date. For the matched individuals of each drug candidate, the Cox proportional hazards regression model was used to calculate hazard ratio and P value dependent on drug use.

*Alzheimer’s Disease Claim Count Analysis*

In this analysis, only members that have made at least two claims for Alzheimer’s Disease were included. Furthermore, we set a threshold for the minimum number of coverage months before and a minimum number of months after the first AD claim was made. Only members that had more coverage than the set thresholds were then considered for this analysis.

Our reason for these filters is consistency and reliability of data. As mentioned previously, the disease and drug history prior to AD diagnosis must be derived from the coverage history itself. If prior coverage history is too short, the data would be insufficient to distinguish drug users from non-users, as well as properly match up pairs during propensity matching analysis.

For post-AD coverage, the variability of range complicates the claim counting process; justification for the threshold is discussed later in this section.

For the claim count analysis, the coverage history sorting and propensity matching procedure is mostly similar to the methodology conducted in the AD incidence analysis as described in Figure S1, with a few key differences, highlighted in Figure S3. The differences are: (1) during the projection of the drug user range (coverage prior to drug use) onto the non-user range, if the non-user’s overall range prior to AD diagnosis is larger than that of the current drug user’s range, the range is projected multiple times onto the non-user’s coverage history up until their first AD claim, with a 4 month interval difference between the projections, and (2) if one projected range from the non-user matches with the range for a drug user, the non-user and all projected ranges from the same non-user are removed from the pool. These differences better encompass the variability in coverage length, as well as the reduced overall pool of applicable members, in the AD-only count analysis compared to the incidence analysis.


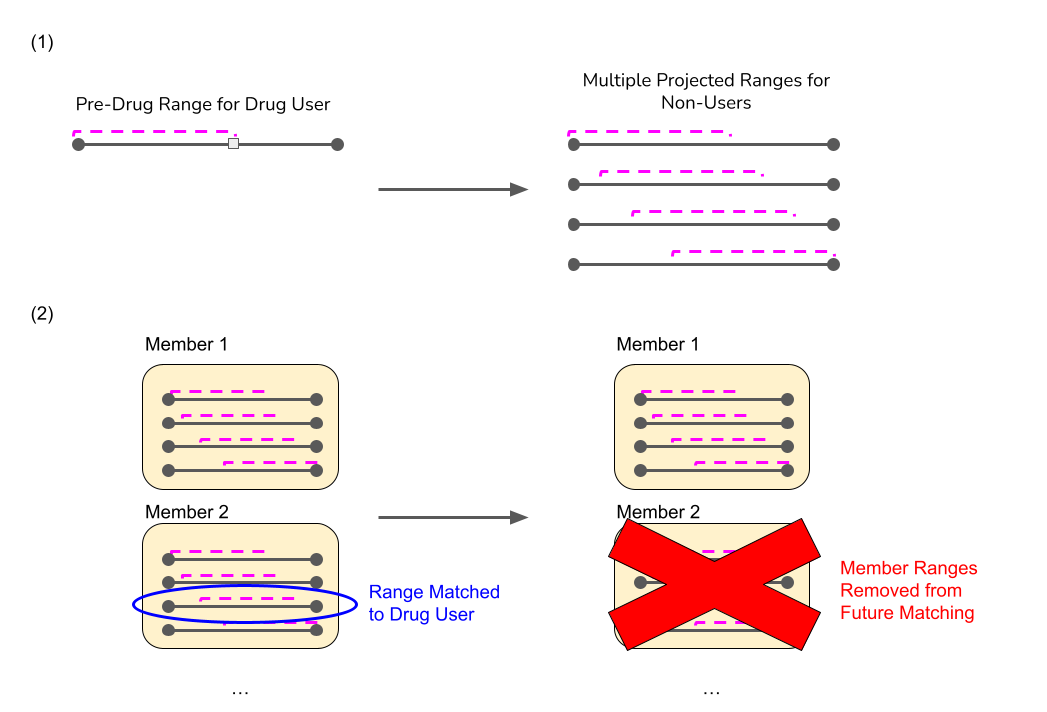

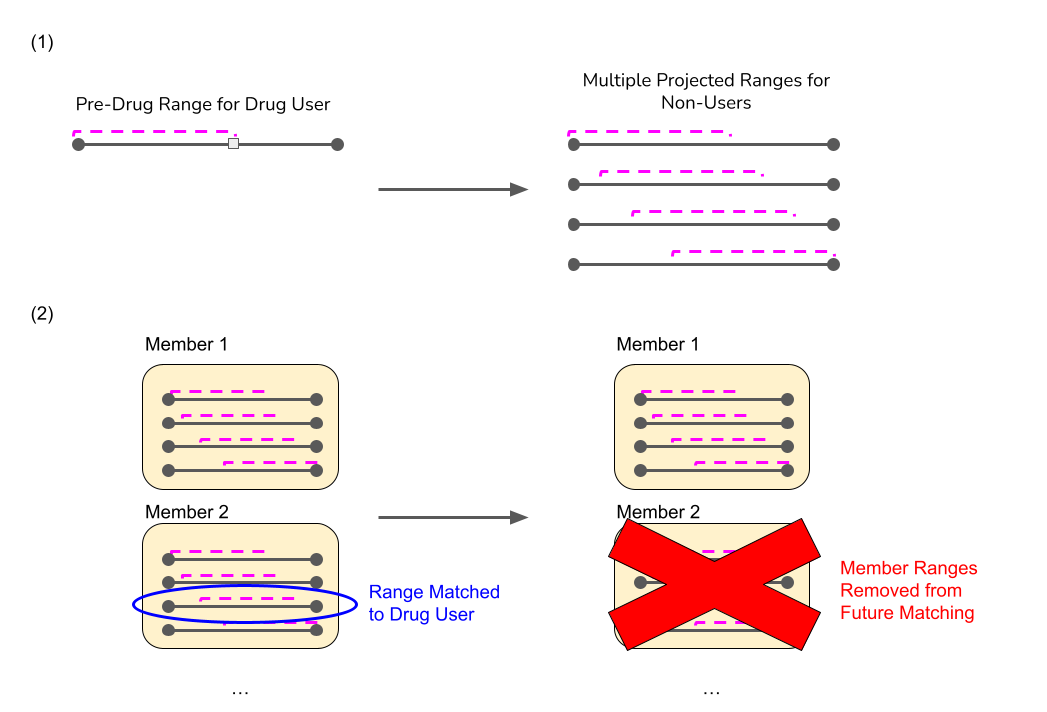


Figure S3. Selection of segmented portions from non-users based on coverage during the claim count analysis. (1) Pre-Drug coverage range of the drug user is projected onto the non-user’s range at multiple intervals if the non-user’s range is greater than the pre-drug range of the drug user. (2) After propensity matching, if one range interval from the non-user is matched, during the next iteration, all segments from the same non-user are removed from the pool such that the non-user is not matched up twice.

Upon selection of matched individuals, the number of AD claims was counted for each member for a set number of months after the member’s first AD claim. Post-AD coverage length may vary between members, and rate of claim filing may also change depending on time since first diagnosis. A direct count of claims would result in bias dependent on coverage length. However, there is a guaranteed minimum number of months after AD diagnosis based on the threshold previously set. For the analysis, then, we count the number of additional AD claims made between onset and the threshold value. From the counts, the paired t-test and p values were calculated between the drug user and non-user groups to obtain an approximated difference in the number of AD claims. By setting a minimum threshold of coverage and then counting only the claims within the threshold, bias is reduced by only checking the claims at a coverage range consistent with all individuals being observed.
