# Supplementary material for: Association study between drug prescriptions and Alzheimer’s disease claims in a commercial insurance database
Source: Alzheimers Res Ther. 2023 Jun 24;15:118. doi: 10.1186/s13195-023-01255-0 (PMC10290352; doi:10.1186/s13195-023-01255-0)
Supplement: Supplementary file 2 — Additional file 2: Supplementary Results. Figure 1. Log 10 Hazard Ratio Distribution Graph from the survival analysis of partial coverage individuals. Supplementary Results Figure 2. Log 10 Hazard Ratio Cumulative Graph from the survival analysis of partial coverage individuals. Supplementary Results Figure 3. QQ plot for raw P values from the survival analysis of partial coverage individuals. [file 13195_2023_1255_MOESM2_ESM.docx]

**Supplementary Results**

*Alzheimer’s Disease Incidence Rate and Survival Analysis for Members with Relaxed Coverage Restrictions*

In consideration for survival bias, the same analysis was conducted on the insured population with allowance for partial coverage of at least 6 months and up to 9 years (full coverage), with the coverage start date set to January 1st, 2012 (the beginning of the database entries). The age restriction including members only of age 65 or older at midpoint (approximately 2016) was retained. This resulted in a population of 4495 members with at least 2 AD claims, and 515004 members without any AD claims.The inflation factor is 16.97. The median value for the hazard ratio is 0.865.

The full table is presented as : “AD_Incidence_Results_PartCoverage_ALL.csv” . Plots related to the survival analysis of the results are shown below.


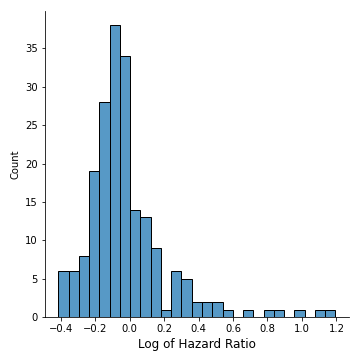


Supplementary Results Figure 1. Log 10 Hazard Ratio Distribution Graph from the survival analysis of partial coverage individuals.


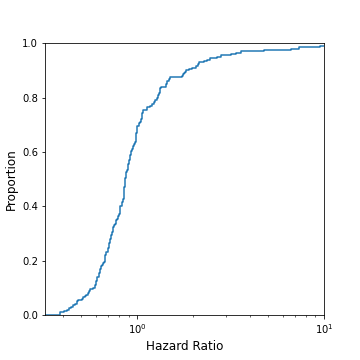


Supplementary Results Figure 2. Log 10 Hazard Ratio Cumulative Graph from the survival analysis of partial coverage individuals.


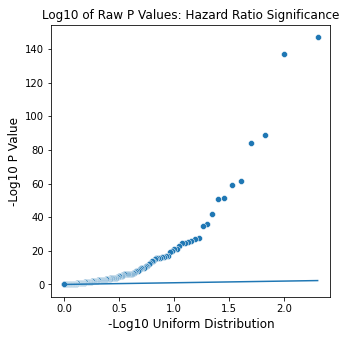


Supplementary Results Figure 3. QQ plot for raw P values from the survival analysis of partial coverage individuals.
